# Supplementary material for: Decelerated Epigenetic Aging in Long Livers
Source: Int J Mol Sci. 2023 Nov 28;24(23):16867. doi: 10.3390/ijms242316867 (PMC10707056; doi:10.3390/ijms242316867)
Supplement: Supplementary file 1 [file ijms-24-16867-s001.zip › Supplementary Table S2.pdf]

Supplementary Table S2. List of PCR primers, pyrosequencing primers and sequences for analysis

Gene	Primer description	Primer sequence	Ta	Sequence to analyze
ASPA	Forward<br>Reverse<br>Pyrosequencing	Biotin-ATTATTTGGTGAAATGATT<br>CAACCCTATTCTCTAAATCTC<br>CCCTATTCTCTAAATCTCA	52°C	ACR1CCATTCTCTAACCAAT ACTTAACCAAAAATACTCCR 2ATTAACT
EDARADD	Forward<br>Reverse<br>Pyrosequencing	TTGGTGATTAGGAGTTTTAGTGTTTT<br>Biotin-CCACCTACAAATTCCCCAAA<br>GGAGTTTGTTATGGAAGAAGTAATA GATTG	56°C	YG1AGAAGATGTTYG2TTGG
ELOVL2	Forward<br>Reverse<br>Pyrosequencing	Biotin-AGGGGAGTAGGGTAAGTGAGG<br>AACAAAACCATTTCCCCCTAATAT<br>ACAACCAATAAATATTCCTAAAACT	60°C	CCR1TGAAACR2TTGAAGAC CR3CCR4CR5CR6AAACCR7A C
PDE4C	Forward<br>Reverse<br>Pyrosequencing	AGGTTTGTAGTAGGTTGAG<br>Biotin-AACTCAAATCCCTCTC<br>GTTATAGTATGATTAGAGTTT	53°C	YG1AAGTATTTGTGGYG2GT AATTTYG3GYG4TTTTATT YG5TATTTAATAGYG6TTTTT ATTYG7GATTYG8GATAAG
