# Supplementary material for: Decelerated Epigenetic Aging in Long Livers
Source: Int J Mol Sci. 2023 Nov 28;24(23):16867. doi: 10.3390/ijms242316867 (PMC10707056; doi:10.3390/ijms242316867)
Supplement: Supplementary file 1 [file ijms-24-16867-s001.zip › Supplementary Table S3.pdf]

Supplementary Table S3. Genomic coordinates of CpG sites of genes included in the study

Gene	№ CpG	Chromosome location (GRCh38)
Aspartoacylase (ASPA)	1	Chr17: 3,476,273
	2	Chr17: 3,476,237
EDAR associated death domain (EDARADD)	1	Chr1: 236,394,382
	2	Chr1: 236,394,370
ELOVL fatty acid elongase 2 (ELOVL2)	1	Chr6: 11,044,661
	2	Chr6: 11,044,655
	3	Chr6: 11,044,647
	4	Chr6: 11,044,644
	5	Chr6: 11,044,642
	6	Chr6: 11,044,640
	7	Chr6: 11,044,634
Phosphodiesterase 4C (PDE4C)	1	Ch19: 18,233,106
	2	Ch19: 18,233,092
	3	Ch19: 18,233,083
	4	Ch19: 18,233,080
	5	Ch19: 18,233,071
	6	Ch19: 18,233,059
	7	Ch19: 18,233,049
	8	Ch19: 18,233,043
